# Contemporary Diagnosis, Management, and Early Outcomes in Children with Kawasaki Disease in Romania: A Single-Center Experience

**DOI:** 10.3390/diagnostics15060656

**Published:** 2025-03-07

**Authors:** Cristina Ramona Rădulescu, Anca Cristina Drăgănescu, Diana Maria Băncilă, Anuţa Bilaşco, Mihai-Rareş Bădescu, Doina Anca Pleşca

**Affiliations:** 1 Department of Pediatrics, “Carol Davila” University of Medicine and Pharmacy, 050474 Bucharest, Romania; cristina.radulescu@umfcd.ro (C.R.R.); diana-maria.slavu@drd.umfcd.ro (D.M.B.); anuta.bilasco@drd.umfcd.ro (A.B.); doina.plesca@umfcd.ro (D.A.P.); 2 “Prof. Dr. Matei Balş” National Institute of Infectious Diseases, 021105 Bucharest, Romania; mihai-rares.badescu@rez.umfcd.ro; 3 “Dr. Victor Gomoiu” Clinical Children’s Hospital, 022102 Bucharest, Romania

**Keywords:** Kawasaki, prolonged fever, IVIG, aspirin, echocardiography, coronary artery lesions, Romania

## Abstract

**Background:** Kawasaki disease (KD) is an acute inflammatory vasculitis with a particularly high incidence of coronary artery complications and constitutes a significant cause of acquired heart disease in children and young adults. **Methods:** We conducted a retrospective analysis of consecutive patients aged 0–18 years hospitalized at the “Prof. Dr. Matei Balş” National Institute of Infectious Diseases in Bucharest with Kawasaki disease over a period of 6 years (2018–2023). **Results:** A total of 25 children were discharged from hospital with this diagnosis during the analyzed period. The mean age was 2.9 years, and 56% were boys. Fever ≥5 days was present in all cases, and the most frequent additional sign was the presence of oral changes. Patients were treated according to in-effect guidelines with intravenous immunoglobulin (IVIG) (100%) and acetylsalicylic acid (68%). Only two cases were considered IVIG resistant and received a second IVIG infusion. Only mild cardiovascular changes were noted in echocardiography: mild coronary artery dilatation (21.7% of cases), mild valvular regurgitation, and small pericardial effusion. Infants displayed less inflammation and higher percentages of leukocytosis, developed an increase in platelet count sooner, received IVIG faster, and had longer hospital stays. Outcomes were generally favorable, and 92% of children were discharged, while the two remaining patients were transferred to other centers. No deaths were recorded. **Conclusions:** To our knowledge, this is the largest contemporary Romanian cohort of Kawasaki disease published to date, outlining the local diagnostic process, therapeutic strategies, and early outcomes of Kawasaki disease.

## 1. Introduction

Kawasaki disease (KD) is an acute inflammatory vasculitis affecting medium-sized vessels, with a particularly high incidence of coronary artery complications [1,2]. It is frequently described as a self-limited febrile illness of unclear etiology [1,3] encountered in childhood, mainly under the age of 5. A geographic (and ethnic) variation of its incidence has been reported, with more cases encountered in eastern Asia [4] and, while it remains a rare disease, there has been a trend towards an increase in the number of cases after the year 2000, followed by a significant reduction during the COVID-19 pandemic [3]. Robust data with respect to the exact incidence in Romania is not available, with only a short series [5] and some sporadic, atypical cases [6,7,8,9] being reported in recent literature.

Despite being acknowledged as self-limited, Kawasaki disease is a significant cause of acquired heart disease both in children and in young adults [10,11].

Found to be in many ways similar to the newly described entity of multisystem inflammatory syndrome in children (MIS-C)/pediatric inflammatory multisystem syndrome (PIMS-Ts), to which it has been compared in numerous accounts, these are in fact now considered to be distinct disorders with some overlapping features [12,13].

The aim of this study was to explore the population characteristics, diagnosis, management, and outcomes of children with Kawasaki disease in a contemporary Romanian cohort by applying in effect guidelines and international recommendations to local cases.

## 2. Materials and Methods

### 2.1. Study Population and Design

We conducted a retrospective study of consecutive patients aged 0–18 years hospitalized at the “Prof. Dr. Matei Balş” National Institute of Infectious Diseases (NIID) in Bucharest, Romania, with Kawasaki disease. The records were studied for a period of 6 years (2018–2023).

The NIID approved this research under a broad regulatory protocol that allowed for analysis of limited patient-level data (C10823/23 October 2024). As such, informed consent was waived.

### 2.2. Data Collection

Hospital electronic records were searched for the keyword “Kawasaki” as the discharge diagnosis. Additional diagnoses, demographics, comorbidities, laboratory measurements, imaging, procedures, and outcomes (death, need for intensive care unit, length of hospital stay (LOS), transfer to a different unit, and hospital discharge) were collected from hospital records. Other variables of interest included clinical presentation and history, evidence of cardiac involvement, and treatment (type, duration, and response). Any previously recorded illness was further subcategorized into chronic disease (including, but not limited to, diabetes, arterial hypertension, obesity and malnutrition, chronic respiratory disease, heart disease, renal disorders, neurodevelopmental conditions and epilepsy, autoimmune and metabolic disorders, and hematologic and oncologic disorders) and non-chronic disease (mainly previous infections, including SARS-CoV-2). Laboratory tests consisted of, but were not limited to, tests that are compatible with a KD diagnosis (as detailed in the definition below), septic and viral screening tests, cardiac biomarkers, and, starting in 2019, diagnostic testing for SARS-CoV-2. With respect to imaging, there was a special focus on echocardiography (including timing and number of studies), but other types of imaging were also included in the dataset provided they had been performed (chest radiograph, abdominal ultrasound, etc.).

Based on the data retrieved, the main diagnosis recorded in the electronic records (either complete KD or incomplete KD) was compared with the diagnostic criteria from international scientific statements for all patients identified in this manner.

Diagnosis of Kawasaki disease according to the 2017 scientific statement of the American Heart Association [1] was maintained in the 2024 update [14]:

Complete KD—fever ≥ 5 days and at least 4 of the 5 principal clinical features:

- Erythema and cracking of lips, strawberry tongue, or erythema of oral and pharyngeal mucosa;
- Bilateral bulbar conjunctival injection without exudate;
- Rash—maculopapular, diffuse erythroderma, or erythema multiforme-like;
- Erythema and edema of the hands and feet in the acute phase (periungual desquamation in the subacute phase);
- Cervical lymphadenopathy, ≥1.5 cm in diameter, usually unilateral.

Incomplete KD—any infant or child with prolonged unexplained fever (≥5 days), fewer than 4 of the principal clinical findings (2 or 3), and compatible laboratory findings (CRP ≥ 3 mg/dL and/or ESR ≥ 40 mm/h) plus either 3 or more additional laboratory findings (anemia for age, platelet count of ≥450,000 after the 7th day of fever, albumin ≤ 3 g/dL, elevated ALT level, WBC count ≥ 15,000/mm^3^, and urine ≥ 10 WBC/hpf) or compatible echocardiographic findings (Z score of left anterior descending coronary artery or right coronary artery ≥ 2.5, coronary artery aneurysm observed, or ≥3 other suggestive features, including decreased left ventricular function, mitral regurgitation, pericardial effusion, or Z scores in left anterior descending coronary artery or right coronary artery of 2 to 2.5).

### 2.3. Statistical Analysis

Comparative data analysis was performed for patients representing a high-risk group (for IVIG resistance/developing coronary artery lesions in particular) aged below 12 moths [15,16], typical Kawasaki cases (aged 1 to 5 years), and older children (above 5 years of age). Similar comparisons have been made by other groups [17]. While some risk scores define age below 6 months as a risk factor [18,19], there were no children that young in this case series.

Statistical analysis was performed using IBM SPSS 23.0 (IBM Corp, Armonk, NY, USA). Univariable analysis was applied to both continuous and categorical variables. Continuous variables were reported as mean ± standard deviation and/or as median and interquartile range (IQR) when appropriate. Between-group comparisons were performed using either a Chi-square or Student’s *t*-test. Statistics with a 2-sided *p* value < 0.05 indicate significant differences.

## 3. Results

### 3.1. Patient Characteristics

**Figure 1:**
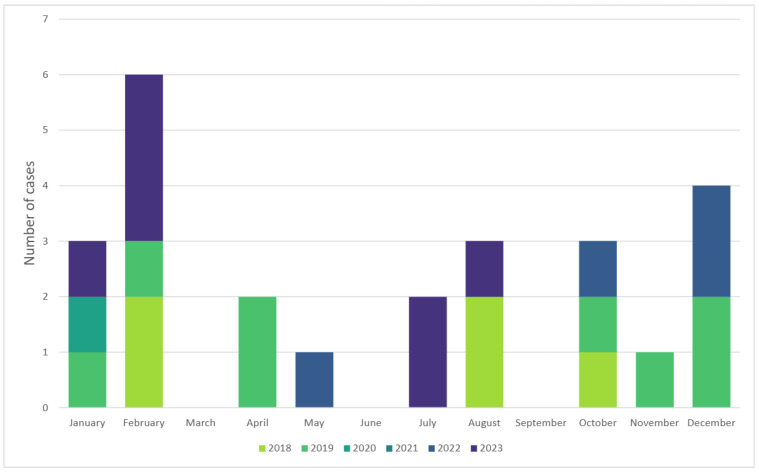
Seasonality of Kawasaki disease showing a peak during cold months.

A total of 33 children had a discharge diagnosis of “Kawasaki” during the analyzed period. Upon reviewing medical charts, eight of these were in fact COVID-19-related cases (MIS-C/PIMS-Ts patients labeled “Kawsaki-like”) and were thus excluded from the analysis. In the remaining group of 25 children the mean age was 2.9 ± 2.17 (range of 7 months–8 years), and 56% were boys. Children aged 1–5 years constituted over half (68%) of the cases. Most children did not suffer from any chronic illnesses, and none had had a previous episode of Kawasaki disease according to records. Only two patients had preexisting (congenital) heart disease: one atrial septal defect and one patent ductus arteriosus. Baseline characteristics are shown comparatively for infants (4 cases), children under 5 (17 cases), and older children (4 cases) in Table 1. There seems to have been a peak in occurrence for KD during the cold months (January in particular) (Figure 1).

### 3.2. Clinical Manifestations

**Figure 2:**
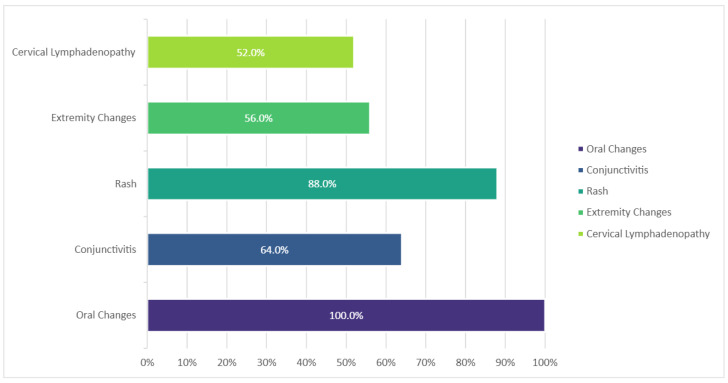
Frequency of main clinical features in KD cases, *n* = 25.

Fever ≥5 days at presentation was noted in all cases. The most frequent additional clinical sign reported was the presence of oral changes (mainly erythema, dryness and cracking of the lips, and diffuse erythema of the oropharyngeal mucosa), followed by the characteristic rash (Figure 2).

### 3.3. Laboratory Findings

All children had some evidence of inflammation (either increased CRP, ESR, or both) and an increased platelet count after the seventh day of fever, and a very high percentage exhibited anemia for their age. Infants developed thrombocytosis sooner than older children, reaching statistical significance compared to the 1–5 years age group (*p* = 0.011), although absolute maximum values were comparable. They also displayed less inflammation but higher percentages of leukocytosis. A summary of relevant laboratory findings may be found in Table 2.

**Figure 3:**
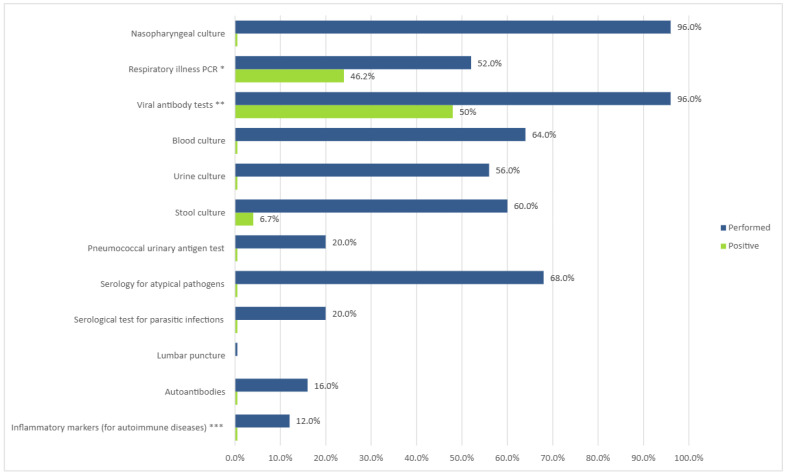
Evaluation of prolonged febrile illness. Values expressed as percent of cases tested and diagnostic yield (percent of positives from number of tests). * Viruses: adenovirus; coronavirus 229; coronavirus HKU1; coronavirus NL63; coronavirus OC43; MERS-CoV; SARS-CoV-2; human metapneumovirus; human rhinovirus/enterovirus; influenza A and B; parainfluenza virus 1, 2, 3, and 4; respiratory syncytial virus. Bacteria: Bordetella parapertussis (IS1001), Bordetella pertussis (ptxP), Chlamydia pneumoniae, Mycoplasma pneumoniae. ** Cytomegalovirus IgM, IgG; Epstein–Barr IgM; adenovirus IgM, Coxsackie virus IgM; urlian IgM; echovirus IgM; herpes simplex virus 1 + 2 IgM, HIV 1 + 2; parvo B19 virus IgM; morbillivirus IgM, IgG; rubella IgM, IgG; SARS-CoV-2. *** IL-1, IL-6, PAI-1, and TNF-alpha.

Most patients were extensively evaluated for their febrile illness by means of serological tests, polymerase chain reaction (PCR), and various cultures, and in some cases, testing for autoimmune diseases was performed. Tests for infectious diseases and their yield are depicted in Figure 3.

### 3.4. Cardiovascular Changes

A total of 23 out the 25 children (92%) had an echocardiography performed during the initial days of admission and, in some cases, there was also a repeat scan during hospitalization (36%). Nearly half of the examinations were performed after intravenous immunoglobulin (IVIG) had already been administered (47.36%); the average day of illness for scans was 10.52 ± 3.45 (range 6–19 days), around two days (median 1.92 ± 1.78, range 0–6 days) after treatment for those carried out after the IVIG infusion.

**Figure 4:**
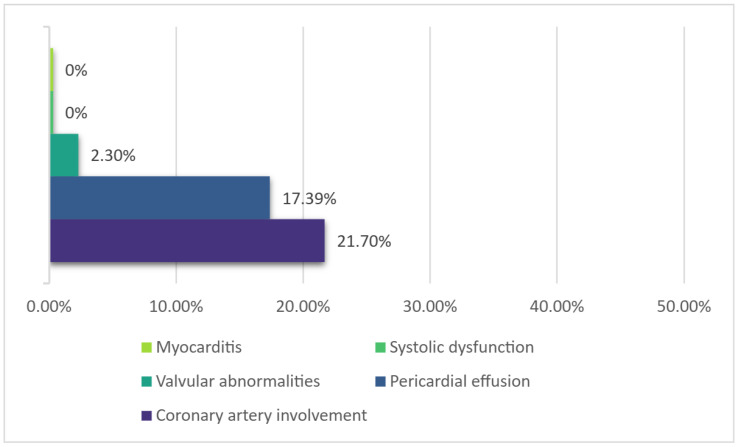
Main cardiovascular changes.

Cardiovascular involvement was mostly mild in nature in this cohort. The most frequent finding was coronary artery involvement (21.7% of cases), all of them exhibiting coronary dilatation only. The presence of coronary artery dilatation did not correlate with the number of days of fever at admission or with the day of fever when IVIG was administered. There were also some patients that displayed mild valvular regurgitation (mostly mitral regurgitation) and a small pericardial effusion (Figure 4).

A diagnosis of myocarditis was not recorded in any of these cases; however, serum troponin was increased in one patient (14.3% of tested patients, 4% overall at a value of 2.94 ng/mL, cutoff of 0.03) and NT proBNP was increased in five, amounting to 55.5% of tested cases and 20% overall, at a mean of 353.98 ± 503.59 ng/L (Table 3). There were no cardiac enzymes measurements for the two CHD children. Neither troponin nor NT-proBNP levels correlated significantly with either coronary artery dilatation or the presence of a pericardial effusion, although there was a significant association between NT-proBNP levels and the presence of valvular regurgitation (r(7) = 0.847, *p* = 0.004). No children exhibited symptoms of heart failure. The ejection fraction was constantly reported as normal, and no wall motion abnormalities were noted. ECGs were performed in 32% of cases; no significant changes were recorded. Cardiac magnetic resonance was not performed.

### 3.5. Other Tests

Various other tests (such as an abdominal ultrasound) and multidisciplinary evaluations (pediatric cardiology, immunology, neurology, gastroenterology, otorhinolaryngology, hematology, etc.) were performed when deemed necessary by the attending clinician. Chest radiograph was performed in 76% of cases; in three children the image was compatible with a diagnosis of pneumonia, while the rest showed non-specific changes only.

### 3.6. Diagnosis

After review, all 25 children had a final diagnosis of Kawasaki disease. Of these, 16 (64%) fulfilled the criteria for classic (complete or typical) KD, while the remaining 8 (32%) were incomplete KD according to the algorithm outlined in Section 2 [1,14].

### 3.7. Case Management

The main therapeutic agents employed in the cohort are depicted in Table 4. All 25 patients received intravenous immunoglobulin (IVIG) treatment at a dose of 2 g/kg, on average on the ninth day of fever. In earlier cases, IVIG was administered in a divided dose over several days (usually five), while all remaining patients received a single infusion. Two cases were considered IVIG resistant and received a second IVIG dose of 2 g/kg, and none of the patients received or required other therapies (e.g., infliximab, anakinra, or other agents).

The proportion of children who received acetylsalicylic acid (ASA) was lower overall (68%) at mean doses of 4.4 mg/kg/day (range 2.94–5.0), and of these only 31.2% were initially treated with ASA at anti-inflammatory doses. Other treatments included other antiplatelet agents (such as dipyridamole) (Table 4). Antibiotics (92%; most patients were empirically started on antibiotic therapy pending the results of other tests), and in some cases antivirals (16%, for herpes simplex virus—acyclovir, and influenza—oseltamivir) were also administered.

Treatment was mostly well tolerated, with few adverse reactions recorded. Two children developed significant anemia (hemoglobin dropping from 11.1 to 5.4 g/dL and from 9.6 to 7.4 g/dL, respectively). The first was an episode of a hemolytic anemia occurring in one of the patients deemed IVIG-resistant. The drop in hemoglobin arose after the second 2 g/kg IVIG dose. The patient received a red blood cell transfusion with a subsequent significant, persistent rise in red blood cell levels and no other events. The other patient demonstrated slowly worsening anemia, with no evidence of bleeding and no apparent relation to the (single) IVIG infusion, and was eventually transferred to a hematology unit for further evaluation.

### 3.8. Outcomes

There were no deaths. Outcomes were generally favorable, and 92% of children were discharged after a mean length of stay of 11.78 days (range of 6–21 days). The remaining two cases were transferred to other centers for continuation of care. Length of stay was significantly higher for younger children compared to patients aged ≥ 5 years (infants, *p* = 0.008, and children aged 1–5 years, *p* = 0.005) (Table 5), although time to diagnosis or complication rates did not vary significantly between groups.

## 4. Discussion

Despite being a rare disease, Kawasaki disease remains a leading cause of acquired heart disease in the pediatric population. One of the difficulties related to this entity stems from the fact that there is no pathognomonic test to allow for a timely and unequivocal diagnosis; rather, it is a diagnostic of exclusion, supported by a specific set of clinical features and echocardiographic and biological markers. Matters were further complicated after 2019, when the COVID-19 pandemic began and a new, similar disorder emerged. The Kawasaki-like syndrome mostly in the form of PIMS-Ts/MIS-C has been identified as a separate entity, with features overlapping those of Kawasaki disease, starting in 2020 [20,21,22] and, although other forms of presentation are possible, in many cases of PIMS-Ts/MIS-C the clinical picture does resemble KD [23]. There are several definitions for PIMS-Ts/MIS-C, varying slightly between one another [24,25,26]. In many cases, it can be difficult to separate the conditions, as children who have experienced an episode of SARS-CoV-2 infection may still develop Kawasaki disease; similarly, many children with Kawasaki-like syndromes fulfill the criteria for either complete or incomplete Kawasaki disease. In our case, 8 children from the initial group of 33 were in fact considered Kawasaki-like syndromes based on a combination of proof of ongoing or recent SARS-CoV-2 infection in addition to clinical features and laboratory findings resembling those of KD.

In the remaining cases, all children fulfilled the internationally recognized criteria for KD [1,14], either complete or incomplete. All cases had at least 5 days of fever when admitted to the hospital. The most frequent additional clinical sign was the presence of oral changes, with a frequency of 100%, followed by a rash in 88%. On average, diagnosis (assumed on the day of fever when specific treatment (i.e., IVIG) was commenced) was established on the ninth day of fever.

As this is a single-center experience, it is not possible to estimate overall incidence accurately. In this cohort, there was a slight male gender predominance (56%), and over 80% children were aged below 5, in line with existing data from other countries [3,27]. It is difficult to make assumptions with respect to very young children based upon this data: there were no newborns (a finding that can easily be explained, as the Institute does not have a neonatal unit), and the youngest child in this group was 7 months old. Two patients had pre-existing mild congenital heart disease. This amounts to an unusually high rate of 8%. Most international data quote rates of congenital heart disease at birth of nearly 2% [28,29], an increase from around 1% in previous studies. Local research mentions similar rates [30], although one paper from a tertiary center that deals with CHD children in particular found higher numbers (5.23–11.47%) [31]. In our case, this high rate is more likely coincidental, as the total number of patients was small, although selection bias cannot be entirely ruled out.

All patients with KD (either classic or incomplete) received IVIG. The rate of ASA therapy was lower, at nearly 70%, and only one third received an initial course of high- or moderate-dose (anti-inflammatory) ASA. Traditionally, ASA has been administered in anti-inflammatory doses (i.e., moderate dose of 30–50 mg/kg per day in Europe and Japan, and high dose of 80–100 mg/kg per day in the United States) until the patient has been afebrile for 48 to 72 h or until the 14th day of illness, with treatment continuing thereafter with low-dose aspirin (3–5 mg/kg per day, i.e., the antiplatelet dose) once a day until 6 to 8 weeks after the onset of illness [1]. However, several recent publications have advocated for the non-inferiority of beginning treatment with the antiplatelet dose [32,33]. While administering low-dose aspirin directly in an effort to avoid potential ASA-related complications, such as Reye syndrome, has become standard practice in some centers, the same cannot be said about omitting ASA treatment altogether, and KD guidelines continue to recommend its use [1,14,34,35]. However, a meta-analysis [36] found that treatment without aspirin was non-inferior to other regimens irrespective of dose (and may also slightly reduce the risk of developing coronary artery lesions when conducted in conjunction with the standard dose of 2 g/kg IVIG). This option was explored in a recently published retrospective study, which also found no additional complications when not using aspirin in the acute phase of KD [37]. When, for various reasons, aspirin is not used, the Italian guideline [34] suggests replacing it with either dipyridamole, ticlopidine, or clopidogrel, while the recent American Heart Association’s scientific statement [14] suggests clopidogrel or dipyridamole. In our cohort, four patients received dipyridamole, either due to hospitalization during influenza outbreaks (a significant number of which were also managed here) or exposure to varicella and the ensuing higher risk of Reye syndrome, or intolerance or allergic reactions to ASA.

The number of serious complications, cardiovascular or otherwise, was low in our cohort. Part of this can be explained by the fact that severely ill, in-shock, or near-shock children may have been admitted to a multidisciplinary center directly. Only mild coronary dilatation was noted in this small group (unfortunately, in many cases, conclusions only rather than exact measurements and Z-scores were documented in the available records). As such, no children were administered more potent antiplatelet agents (such as clopidogrel) or an anticoagulant. The rate of coronary artery dilatation occurrence appeared to be quite high for a population treated with IVIG (21.7% of children). However, data on coronary artery lesions (CALs) mentioned in guidelines [1] (23% without IVIG, 8% with 4 infusions of low-dose IVIG, 4% (4.9%) with single high-dose IVIG) refer to scans performed either at 2 weeks [38] (with a reported prevalence of 8%) or at 30 or 60 days of illness [39] (with a reported prevalence of 10.2% and 4.9%, respectively). In this cohort, scans were performed earlier—on average, on day 10 or 11 of illness (range 6–19)—which makes comparison difficult. A recent paper [40] found a prevalence of CALs (any type) detected at the initial echocardiography of 5.3% on day 7 and 14.1% on day 10, increasing to 24.9% on days ≥ 11. Figures also vary according to the criteria used to delineate CALs (i.e., the older Japanese Ministry of Health dichotomous criteria [41] or according to Z scores), with published papers reporting a higher overall incidence of CALs using the Z-score system, nearing 40% in some studies [42,43]. Coronary artery aneurysm (CAA) formation is a significant parameter, since there appears to be an increased risk of post-discharge cardiovascular events after KD, and it has been shown that this risk is higher in patients with aneurysms [10]. Aneurysms were not found in this cohort.

No acute cardiovascular events were noted. Although a minority of children exhibited an increase in cardiac biomarkers, this did not translate into symptoms or impaired cardiac function as assessed by echocardiography. There was no apparent correlation between this increase and other changes, such as coronary artery dilatation; however, the number of tests was objectively small. Patient testing increased over time, reflecting a change in local protocols. NT-proBNP levels were found to be higher in the presence of valvular (in this case mitral) regurgitation. Increasing NT-proBNP levels according to mitral regurgitation severity has long since been demonstrated in adults [44,45] and has also been proven to be of use in the grading and timing of intervention in children [46,47]. In this case, however, the association should be treated with caution, as it resulted from a single patient with a significant increase in NT-proBNP (Table 3). The mitral regurgitation was quantified as mild only, and LV dilatation was not recorded. Other papers have pointed to an increase in BNP and/or NT-proBNP levels in acute pediatric inflammatory diseases (including KD, among others) while discussing various possible underlying mechanisms for this increase [48], whereas some authors have suggested using NT-proBNP as an additional diagnostic marker for suspected incomplete KD [49,50]. While other factors may also contribute to an increase in NT-proBNP levels in acute inflammatory diseases, since at least some of the patients enrolled in these studies had echocardiographic changes compatible with increased LV filling pressures and wall stress, it is difficult to make a clear distinction among probable causes.

Although follow-up visits were performed, echocardiography data from these visits were not accessible; it is therefore not possible to assess mid- and long-term cardiovascular response and complications.

IVIG resistance was low in this cohort (found in only 2 children, 8% of those treated). Several scoring systems have been developed in an attempt to predict IVIG resistance [15,16,18,51]. The American College of Rheumatology/Vasculitis Foundation Guideline [35], while acknowledging these risk scores, defined age < 6 months and a Z score of ≥2.5 for either the left anterior descending or right coronary artery at the time of the initial echocardiography as high-risk features (for IVIG resistance/CAAs) and suggests employing IVIG with adjunctive glucocorticoids or with other non-glucocorticoid immunomodulatory immunosuppressive agents as initial therapy in these patients. The very recent update on the diagnosis and management of Kawasaki disease from the American Heart Association [14] employs the same criteria in conjunction with the Son risk score [19], which has been validated in a North American population. None of the children in this cohort with IVIG resistance exhibited high-risk features according to these criteria.

A significant number of children received antibiotics; however, these were mostly started empirically before the diagnosis of KD was established. In addition, some children in this cohort received small doses of steroids for various reasons before a final diagnosis was reached.

There were no major differences between age groups, most likely due to the small sample size. However, infants displayed a trend toward less inflammation and higher percentages of leukocytosis, developed an increase in platelet count sooner, received IVIG faster, and had significantly longer hospital stays, possibly due to having more complex nursing needs.

One of the main limitations of this study is that it represents a single center’s experience. Data presented here may not necessarily reflect national circumstances, and mid- and long-term outcomes could not be assessed. A collaborative effort of multiple centers would be appropriate to provide a more accurate perspective. In addition, the retrospective nature of the study meant that certain data were either missing or could not be retrieved, potentially weakening inferences. The NIID is a facility specializing in the evaluation and treatment of severe and/or difficult cases of infectious diseases (either diagnosed as such or assumed to be); severe, unstable children, as well as very young children (especially neonates), will most likely be admitted somewhere else. On the other hand, hospitalization here translates to a generally extensive work-up for other types of febrile illnesses, making an alternative diagnosis implausible and thus providing good-quality data on this specific topic.

## 5. Conclusions

To our knowledge, this is the largest contemporary Romanian cohort of Kawasaki disease published to date. While the total number of cases is small, data analysis allowed for reasonable characterization of the diagnostic process, therapeutic strategies, and early outcomes. The study has identified several characteristics of the local care continuum and should permit an improvement in this process.

## Figures and Tables

**Table 1:** Baseline characteristics: population characteristics and relevant medical history.

Variable	Overall	Age < 1	Age 1–5	Age ≥ 5
Patient characteristics				
Male sex	14/25 (56%)	4/4 (100%)	9/17 (52.9%)	1/4 (25%)
Living in an urban area	16/22 (72.7%)	2/3 (66.7%)	10/15 (66.7%)	4/4 (100%)
Medical history				
Non-chronic disease	18 (78.3%)	3/4 (75%)	13/16 (81.3%)	2/3 (66.7%)
Chronic disease	4 (17.4%)	1/4 (25%)	3/16 (18.8%)	-
CHD	2 (8.7%)	-	2/16 (12.5%)	-

Values expressed as absolute number out of total number of available data points in the respective category, and valid percentage. CHD, congenital heart disease.

**Table 2:** Main laboratory findings.

Parameter	Overall	Age < 1	Age 1–5	Age ≥ 5
Inflammation				
CRP ≥ 3 mg/dL	24/25 (96%)	3/4 (75%)	17/17 (100%)	4/4 (100%)
ESR ≥ 40 mm/h	21/22 (95.5%)	2/3 (66.7)	15/15 (100%)	4/4 (100%)
Other findings				
Anemia for age	24/25 (96%)	4/4 (100%)	16/17 (94.1%)	4/4 (100%)
Maximum platelet count (PLT max)	844.88 ± 200.98	873.5 ± 150.65	879.41 ± 211.27	669.50 ± 116.34
Day of fever when PLT max	13.2 ± 3.47	9.5 ± 3.51	14.29 ± 2.97	12.25 ± 3.30
PLT count of ≥450,000 after the 7th day of fever	25/25 (100%)	4/4 (100%)	17/17 (100%)	4/4 (100%)
Albumin ≤ 3 g/dL	5/19 (26.3%)	1/2 (50%)	4/14 (28.6%)	-
Elevated ALT level *	16/24 (66.7%)	1/4 (25%)	5/16 (31.3%)	2/4 (50%)
WBC count ≥ 15,000/mm^3^	20/25 (80%)	4/4 (100%)	13/17 (76.5%)	3/4 (75%)
Urine ≥ 10 WBC/hpf	12/23 (52.2%)	2/4 (50%)	9/15 (60%)	1/4 (25%)

Data provided as mean and standard deviation, and as absolute number out of total number of available data points in the respective category and valid percentage, as appropriate. CRP, C-reactive protein; ESR, erythrocyte sedimentation rate; PLT, platelet(s); ALT, alanine transaminase; WBC, white blood cell; hpf, high power field. * Defined as over 45 U/L as per local laboratory normal values.

**Table 3:** Cardiac biomarker levels and main cardiovascular changes.

	Troponin (ng/mL)	NT-proBNP (pg/mL)	Coronary Artery Dilatation	Valvular Regurgitation	Pericardial Effusion
Patient 1	**2.94**	40.8	−	−	−
Patient 2	<0.012	**1492**	−	+	−
Patient 3	0.00	0.0	−	−	−
Patient 4	0.00	**387**	−	−	−
Patient 5	0.03	78	−	−	−
Patient 6	0.03	**833**	+	−	−
Patient 7	0.03	**>125**	−	−	−
Patient 8	0.03		−	−	+
Patient 9	0.03	**267**	−	−	−
Patient 10		88	−	−	−

+, Present; −, absent; tab left blank, test was not performed. Values above assay cutoff levels are displayed in bold. NT-proBNP, N-terminal prohormone of brain natriuretic peptide.

**Table 4:** Therapy during acute illness.

Medication	Overall	Age < 1	Age 1–5	Age ≥ 5
Intravenous immunoglobulin *n* (%)	25 (100%)	4 (100%)	17 (100%)	4 (100%)
Day (of fever) of administration	9.09 ± 3.25	7.33 ± 1.15	9.07 ± 2.15	10.50 ± 6.76
Second dose	2 (8%)	-	2 (11.8%)	-
Acetylsalicylic acid *n* (%)	17 (68%)	3 (75%)	11 (64.7%)	3 (75%)
Other antiplatelet or anticoagulant agents *n* (%)				
Dipyridamole	4 (16%)	1 (25%)	3 (17.6%)	-

Data provided as mean and standard deviation, and as absolute number and valid percentage, as appropriate.

**Table 5:** Hospital admission attributes.

Variable	Overall	Age < 1	Age 1–5	Age ≥ 5
Length of hospital stay * (days)	11.78 ± 3.6	12.5 ± 2.38	12.63 ± 3.32	6.33 ± 0.58
Transfer to multidisciplinary center	2 (8%)	-	1 (5.9%)	1 (25%)

Values expressed as mean and standard deviation, and as absolute number and percentage, as appropriate. * Excluding the two cases that were transferred to other hospitals.

## Data Availability

The datasets generated and analyzed in this study are available on request from the corresponding author due to ethical and/or legal reasons.

## Abbreviations

The following abbreviations are used in this manuscript:

KD	Kawasaki disease
IVIG	Intravenous immunoglobulin
MIS-C	Multisystem inflammatory syndrome in children
PIMS-Ts	Pediatric inflammatory multisystem syndrome
NIID	“Prof. Dr. Matei Balş” National Institute of Infectious Diseases
LOS	Length of hospital stay
CRP	C-reactive protein
ESR	Erythrocyte sedimentation rate
PLT	Platelet(s)
ALT	Alanine transaminase
WBC	White blood cell
hpf	High power field
CHD	Congenital heart disease
NT-proBNP	N-terminal prohormone of brain natriuretic peptide
ASA	Acetylsalicylic acid
CALs	Coronary artery lesions
CAA	Coronary artery aneurysm
